# Supplementary material for: Fabrication of a Polybutylene Succinate (PBS)/Polybutylene Adipate-Co-Terephthalate (PBAT)-Based Hybrid System Reinforced with Lignin and Zinc Nanoparticles for Potential Biomedical Applications
Source: Polymers (Basel). 2022 Nov 22;14(23):5065. doi: 10.3390/polym14235065 (PMC9739168; doi:10.3390/polym14235065)
Supplement: Supplementary file 1 [file polymers-14-05065-s001.zip › polymers-2021481-supplementary.pdf]

polymers

MDPI

Table S1 Tensile properties of neat biopolymers and their blends.

Sample	Tensile strength at yield (MPa)	Tensile strain at break %	Tensile modulus (MPa)
PBS	37.67 ± 0.83	457 ± 24.02	357 ± 15.47
PBAT	8.38 ± 0.11	1040 ± 9.76	52.01 ± 28.78
PBS-PBAT (90/10)	34.30 ± 0.37	112.94 ± 34.94	341.06 ± 6.93
PBS-PBAT (80/20)	30.52 ± 0.26	195.24 ± 31.63	298.52 ± 4.67
PBS-PBAT (70/30)	26.99 ± 0.32	367.01 ± 86.87	253.49 ± 13.40
PBS-PBAT (60/40)	24.96 ± 0.42	598.03 ± 56.55	186.28 ± 10.26

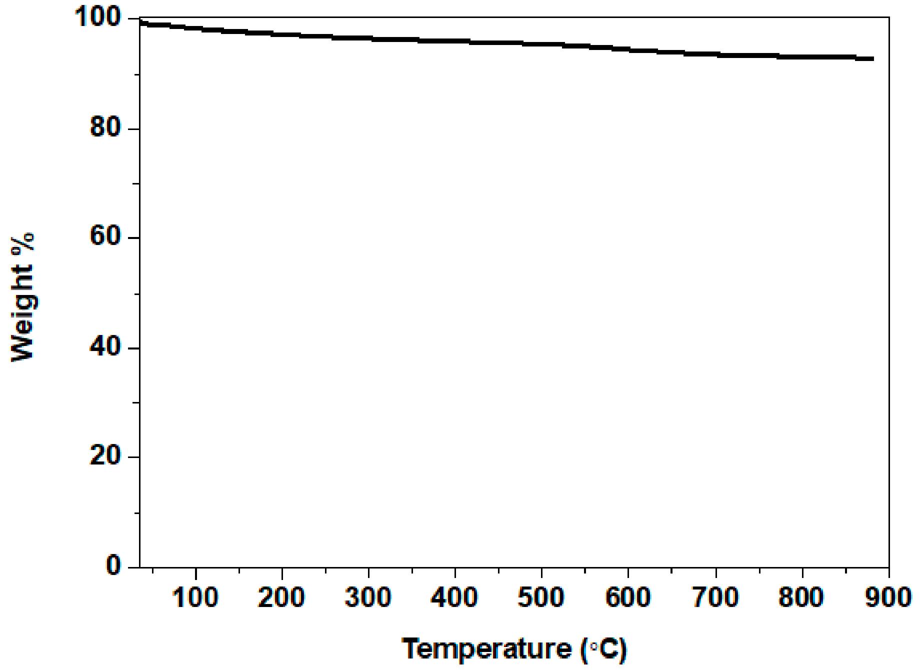

Figure S1 TGA curve of zinc nanoparticles.
